# Economic burden of hemodialysis among patients with chronic kidney disease in Dar es-Salaam region in Tanzania - a cross-sectional study

**DOI:** 10.1186/s12962-026-00749-6

**Published:** 2026-04-30

**Authors:** Grace Mallange, Oddvar Martin Kaarbøe, Alphoncina Kagaigai, Novatus Tesha, Jonathan Mngumi, Jamila Didi, Amani Thomas Mori

**Affiliations:** 1 Muhimbili University of Health and Allied Sciences, P. O. Box 65001, Dar- es-Salaam, Tanzania; 2 Section for Ethics and Health Economics, Department of Global Public Health and Primary Care, University of Bergen, P.O. Box 7804, Bergen, 5020 Norway; 3 Muhimbili National Hospital - Upanga, P.O. Box 65000, Dar-es-Salaam, Tanzania; 4 Muhimbili National Hospital - Mloganzila, P.O Box 65000, Dar-es-Salaam, Tanzania

**Keywords:** Hemodialysis, Patient with chronic kidney disease, CKD, Economic burden, Tanzania

## Abstract

**Background:**

Chronic Kidney Disease (CKD) is a major public health problem with increasing morbidity and mortality, and with high cost to patients and the health care system. This study aims to estimate the patient’s cost, incidence of catastrophic health expenditure, and factors influencing the cost of hemodialysis in Dar es-Salaam region, Tanzania.

**Methods:**

This cross-sectional study included 437 CKD patients who were on hemodialysis in eight centres. Data was collected in two rounds between March and April 2023 and April to May 2024 by interviewing patients or caregivers using a pre-tested questionnaire. Cost data were collected in Tanzanian shillings (TZS) and converted to 2024 US dollars ($), using the exchange rate of 1$ = 2,314 TZS. Data was analyzed with Stata 18. A mixed-effects generalized linear model (GLM) with gamma distribution was used to assess the association of independent variables with hemodialysis cost. The incidence of catastrophic health expenditure (CHE) was calculated as the proportion of households whose annual spending on hemodialysis treatment exceeded 10% or 25% of their average annual household income.

**Results:**

The average annual cost of hemodialysis was $3,099. Direct medical costs contributed 49.1% ($1,523), direct non-medical costs 20.8% ($645), and indirect costs 30.1% ($931). The economic burden was four times higher among patients who solely paid in cash compared to those who were fully insured i.e. $7,9301 versus $1,958. Only 40% of patients reported they were still working after starting hemodialysis; however, on average, they missed about 12 working days every month. Proportion of 0.91 patients incurred annual hemodialysis treatment costs that exceeded 10% of their annual income, while 0.77 patients experienced treatment costs that exceeded 25% of their income. The patients’ cost of hemodialysis was significantly associated with having a monthly income greater than $217, solely paying in cash, receiving full or partial exemption, having partial insurance, undergoing hemodialysis for two to three years, and attending more than one hemodialysis session per week.

**Conclusion:**

The economic burden of hemodialysis is particularly high among patients who pay out-of-pocket compared to those with health insurance coverage, contributing to a significantly higher incidence of catastrophic health expenditure among uninsured patients (91% vs. 77%). Therefore, the government must implement policies that protect patients without health insurance, as they remain at substantial risk of being driven into impoverishment and poverty.

**Supplementary Information:**

The online version contains supplementary material available at 10.1186/s12962-026-00749-6.

## Background

Chronic kidney disease (CKD) is defined as abnormalities of kidney structure or function that last for more than three months and have long-lasting negative health implications [1]. It is a condition in which the kidneys are damaged and do not function properly, resulting in excess fluid and waste retention in the body.

CKD has been identified as a major public health issue worldwide, with increasing mortality and morbidity rates. CKD was reported to be the ninth leading cause of mortality in 2023, with the global age-adjusted prevalence of 14.2% [2]. The prevalence is also reported to be around 15.2%, 16.3%, and 12.2% in the low-, middle- and high-income countries [2]. An earlier meta-analysis study conducted in sub-Saharan Africa in 2014 estimated that the prevalence of CKD was around 13.9% [3]. CKD was reported to account for 2.53% of the total deaths and 1.64% of the total Disability Adjusted Life Years (DALYs) worldwide in 2019 [4].

Apart from mortality and morbidity, CKD has also been reported to cause a high economic burden to patients as well as health systems. In Canada, patients undergoing hemodialysis treatment incur an average cost of between $1,400 and $2,500 annually [5], while in India they incur $5,740.80 ($478.40 monthly) [6], and $7,739.17 in Ethiopia [7]. The average annual direct medical and non-medical costs of hemodialysis were reported at $1612.9 in Burkina Faso [8], $3859.1 in Sudan [9] and $28,280 in the Democratic Republic of Congo [10].

Hemodialysis has been reported to cause catastrophic health expenditure (CHE) to patients and their households. Studies conducted in Korea reported that households with CKD patients had the highest rate of CHE (23%) compared to households with patients with other chronic diseases [11]. India also reported CHE of 96% and 95% for patients with CKD who were undergoing hemodialysis treatment [12, 13]. Factors that were reported to be associated with the cost of hemodialysis include age, occupation, monthly family income, type of health facility, hemodialysis duration, and medical insurance type [7, 8, 14, 15].

Tanzania has also seen an increase in CKD cases, with one study reporting a prevalence of 7% in the Northern part of the country [16] and another reporting a 12.4% prevalence in a rural district in the Coast (Pwani) region [17]. Additionally, CKD accounted for 1.76% of the total deaths and 0.95% of the total DALYs in Tanzania in 2019 [4]. Hemodialysis is the most commonly practiced dialysis modality in Tanzania for patients with CKD. In 2018, there were 28 public and private hemodialysis facilities, including hospitals and stand-alone facilities. Fifteen out of the 28 facilities were in the Dar es-Salaam region, which caters to over 50% of the country’s hemodialysis patients [18].

A study conducted in Tanzania in 2014 estimated the annual costs of hemodialysis from the provider’s perspective to be $27,440 [19]. However, to the best of our knowledge, no research has been undertaken to assess the economic burden of hemodialysis from the patient’s perspective. Therefore, this study aims to address this gap by estimating the average costs incurred by patients undergoing hemodialysis in Tanzania. Additionally, it will identify the factors associated with these costs and the incidence of CHE.

## Methodology

### Study site

This study employs a facility-based costing approach and was conducted in eight out of the 15 hemodialysis facilities in Dar es-Salaam region. The eight facilities were deliberately chosen due to their high number of CKD patients undergoing hemodialysis and their diverse ownership, comprising four public and four private facilities, see Table A1 in the appendix.

### Study design and study population

This cross-sectional study involved 437 (79%) out of 553 registered patients receiving hemodialysis at the selected health facilities during the data collection period. The number of interviewed participants was slightly above the estimated sample size of 427, which was calculated by assuming a 50% prevalence and a statistical power of 95% with a 5% margin of error. For patients under the age of 18 (five patients) or those with critical health issues that prevented them from participating in the survey (25 patients), as advised by the medical staff, their caregivers were asked to participate on their behalf.

### Study perspective

This study adopted the patient’s perspective, encompassing all costs incurred by the patients and their families while seeking hemodialysis. These costs included direct medical expenses (consultation fees, medicines and medical supplies, investigation (laboratory tests) fees, hospitalization, and insurance premiums), direct non-medical expenses (transportation and meal costs), and indirect costs (loss of income due to inability to work, i.e., loss of productivity).

### Data collection and analysis

Data was collected through face to face interviews with CKD patients or caregivers using a pre-tested structured questionnaire created in the Open Data Kit (ODK). Pretesting of the tool was conducted by interviewing 10 CKD patients at one of the dialysis facilities. Adjustment of the questionnaire after pre-testing included improving the flow of the questions and adding questions to characterize their health insurance. The tool was forward-backwards translated from English into Kiswahili (local language). Four research assistants were trained in a four-hour session led by the first author of this study. Data were collected in two rounds between March and April 2023, during which 292 patients were interviewed and between April and May 2024, during which 145 interviews were held. Analysis was conducted in 2024.

We used the Human Capital Approach (HCA), which estimates loss in productivity as a reduction of individual working capacity due to illness. This method assigns monetary value to time lost from work using gross wage, focusing on the impact of disease-related morbidity or mortality on productivity. The loss in productivity (indirect) cost related to morbidity due to illness was estimated using formula below [20, 21].

  

$$\:IC\:=\:\sum\:\:(D_{i}\:\times\:\:W_{i})$$

Where:

IC = Indirect Cost

D_i =_ Number of Workdays lost due to illness

W_i =_ Average daily wage

We did not include the loss of productivity for the caregivers. In instances where income data was unavailable (for both private and government employees), the current national monthly minimum wage of TZS 300,000 ($123.9) for the formal sector was used [22]. Students, retired individuals, and those who reported being unemployed reported zero income, and therefore, no loss in productivity. Some patients reported missing more workdays than they were scheduled to work in a month. For these patients, the number of missed workdays was estimated by considering the scheduled workdays as the missed workdays.

The direct and indirect costs were summed per patient to determine the average annual cost. All costs were converted to US dollars by adjusting Tanzanian Shillings (TZS) to 2024 USD using the prevailing exchange rate of 1$ = TZS 2,314. The incidence of catastrophic health expenditure (CHE) was calculated as the proportion of households whose annual spending on hemodialysis treatment exceeded 10% or 25% of their average annual household income [13, 23–25].

$$\:CHE\:=\:\left(\frac{AHC}{AI}\right)*\:100$$

Where AHC = Average annual hemodialysis cost; AI = Average annual household income. CHE was coded as “1” if it exceeded the10% or 25% thresholds and “0” if otherwise.

A mixed-effects generalized linear model (GLM) with gamma distribution was used to assess the association of independent variables with hemodialysis cost, accounting for the skewness of cost data and controlling for clustering at the facility level [26–28]. Variables that were included in the model were selected based on evidence from previous studies and theoretical relevance to patient-level cost variation. Sociodemographic variables such as age, sex, education, occupation, and income are well-documented predictors of healthcare utilization patterns and financial vulnerability among patients with chronic kidney disease, as shown in studies from Ethiopia, Burkina Faso, Malaysia, and China [7, 8, 14, 15]. Facility ownership, health insurance status, payment modality, and number of weekly hemodialysis sessions directly influence treatment payments and the likelihood of out-of-pocket expenditure. In addition, treatment duration and the presence of comorbidities have been associated with increased clinical complexity and higher treatment needs, which, in turn, affect both direct and indirect costs.

For bivariate analysis, all variables for which data was collected were included i.e., facility ownership, patients’ age, sex, occupation, income marital status, education, number of hemodialysis sessions a patient attends per week, treatment payment modality (how a patient pay for their treatment i.e., have insurance, pay fully in cash, or have full or partial exemption), treatment duration (how long a patient is in hemodialysis treatment) and if a patient had other health comorbidities such as hypertension, diabetes, thyroid diseases etc. Variables with a p-value of less than 0.2 in the bivariate analysis were included in the final multivariate mixed-effects GLM model. A p-value of less than 0.05 was used to identify statistically significant variables using Stata 18.0 software.

### Ethical issues

Ethical approval was obtained from the Institutional Review Board at Muhimbili University of Health and Allied Sciences, Ref no. MUHAS-REC-03-2023-1574). Also, permission to conduct the study was sought from the selected facilities. All participants included consented to participate in the study.

## Results

### Sociodemographic and health service-related characteristics

Table 1 presents the sociodemographic characteristics of the study participants. Nearly 68% of the participants were male, and over 82% were above 41 years of age. The majority (81%) were married, and approximately 78% resided in Dar es-Salaam region before starting hemodialysis.

**Table 1:** Sociodemographic and health service-related characteristics (*n* = 437)

Variable	Frequency (*n*)	Percentage (%)
**Sex**		
Male	297	67.96
Female	140	32.04
**Age (Years)**		
≤ 20	7	1.60
21–40	68	15.56
41–60	190	43.48
≥ 61	172	39.36
**Marital Status**		
Married	354	81.01
Not Married	49	11.21
Widowed	34	7.78
**Place of Residence Before Treatment**		
Dar es Salaam	341	78.03
Other Regions	96	21.97
**Occupation**		
Retired	126	28.83
Self-employed	110	25.17
Public sector employee	73	16.70
Unemployed	63	14.42
Private sector employee	28	6.41
Student	11	2.52
Day laborer	7	1.60
Other	19	4.35
**Income per month**		
$0	149	34.10
$1–$43.2	20	4.58
$43.6–$86.4	32	7.32
$86.9–$129.6	32	7.32
$130.0–$172.8	16	3.66
$173.3–$216.0	32	7.32
≥ $216.5	122	27.92
Missing	34	7.78
**Educational level**		
Higher education	202	46.22
Secondary education	122	27.92
Primary education	102	23.34
No education	11	2.52
**Treatment payment modality**		
Fully insured	342	78.26
Cash	51	11.67
Partial exempted	39	8.92
Partial insured	4	0.92
Fully exempted	1	0.23
**Ownership of the health facility**		
Public	255	58.65
Private	182	41.65
**Length of hemodialysis**		
Less than 1 year	150	34.32
2–3 years months	176	40.27
4 years and above	111	25.40
**Hemodialysis sessions per week**		
1 session	14	3.20
2 sessions	122	27.92
3 sessions	301	68.88
**Presence of comorbidities**		
Hypertension	169	50.69
Cardiovascular diseases	87	28.62
Diabetic	32	10.53
Thyroid diseases	4	1.32
Others (Cancer, HIV, TB)	12	3.95

Regarding occupation, approximately 25.2% were self-employed, and 23.1% were employed in either the government or private sector, while about 29% were retired. Approximately 34% of the participants had an inconsistent source of income, 4.5% had a monthly income of less than $43, and about 28% earned more than $216 per month. Over half (57%) of the participants earned less than the minimum monthly wage in the formal sector. About 23% of participants had at most a primary education, while 46% had at least one year of college education (higher education).

Regarding the funding of hemodialysis, 78.3% of the participants were fully insured, 11.7% paid solely in cash, 8.9% covered costs through partial self-funding with government assistance (partially exempted), 0.23% were fully exempted, and 0.92% were partially insured, i.e., their insurance only paid a share of the cost.

Over half (58.6%) of the participants were receiving treatment at public health facilities. A greater proportion of participants (40%) had been receiving treatment for two to three years, compared to those who had been in treatment for less than one year (34%) and those who had been in treatment for more than four years (25%). Among the study participants, 68.9% attended the required three treatment sessions per week, while 27.9% attended two sessions and about 3.2% one session per week. Additionally, about 80% of the participants had other health comorbidities, including 50.7% with hypertension, 28.6% with cardiovascular diseases, 10.5% with diabetes, 1.3% with thyroid diseases and 1.4% with other conditions such as cancer, AIDS and tuberculosis (TB).

## The cost of hemodialysis

Table 2 illustrates the cost of hemodialysis and their distribution. The mean annual cost of hemodialysis was $3,099.1. Direct medical costs accounted for 49.1% ($1,522.5) while direct non-medical costs and indirect costs constituted 20.8% ($645.1) and 30.1% ($931.4) of the total costs, respectively.

**Table 2:** Average annual costs ($) associated with hemodialysis (*n* = 437)

By cost components	Mean (SD)	Median (IQR)
Consultation	28.8 (142.3)	0 (0–0)
Investigation	0 (0)	0 (0–0)
Medicine and medical supplies	152.9 (713.7)	0 (0–0)
Hemodialysis session	1,007.9 (2657.1)	0 (0–0)
Hospitalization	47.2 (869.9)	0 (0–0)
Insurance premium	285.7 (257.7)	233.4 (0–518.7)
**Sub-total (Direct medical cost)**	**1**,**522.5 (3289.3)**	**432.1 (216.1–649.1)**
Transportation	477.6 (610.2)	337.1 (134.8–898.9)
Meals	167.5 (357.3)	89.9 (44.9–179.8)
**Sub-total (Direct non-medical cost)**	**645.1 (805.4)**	**427.4 (224.7–898.8)**
**Loss in productivity (Indirect cost)**	**931.4 (2343.1)**	**147.4 (147.6–888.9)**
**Average annual cost**	**3**,**099.1 (4287.3)**	**1**,**669.3 (1**,**016.2–3**,**197.8)**

### Cost of hemodialysis by facility type/ownership

Table 3 shows the hemodialysis cost by facility type. Government-owned facilities had a higher average cost ($3413.6) compared to privately owned facilities ($2658.3). The direct medical cost was high in government-owned facilities ($1937.2) compared to privately owned facilities ($941.5). This can be attributed to a high number of fully insured patients in privately owned facilities (92%) as compared to 67% of fully insured patients in government-owned facilities. Direct non medical cost was not very different in both facilities, with $608.6 (government) and $696.3 (private) facilities. Indirect cost was slightly higher in private facilities ($1020.6) compared to government facilities ($867.8).

**Table 3:** Average annual cost of hemodialysis by facility type/ownership

By Facility type	Mean (SD)	Median (IQR)
**Government (*n*** **= 255)**		
Direct medical cost	1937.2 (3945.4)	432.15 (155.6–2022.5)
Direct non medical cost	608.6 (885.3)	332.6 (157.3–808.9)
Indirect Cost	867.8 (1786.8)	147.57(147.6–1037.2)
**Average annual cost**	**3413.6 (4612.3)**	**1937.9 (984.2–3961.4)**
**Private (*n*** **= 182)**		
Direct medical cost	941.5 (1907.0)	432.2 (233.4–649.1)
Direct non medical cost	696.3 (676.8)	449.4 (269.7–898.9)
Indirect cost	1020.6 (2954.9)	147.5 (147.5–770.4)
**Average annual cost**	**2658.3 (3753.6)**	**1399.8 (1020.5 – 2446.8)**

### Cost of hemodialysis by payment modalities

Table 4 shows the cost of hemodialysis by payment modalities. Cost was slightly higher for participants who were partially insured compared to those who paid solely in cash, i.e. $9,985.8 versus $7,930.8. Furthermore, the financial burden was four times higher for participants who solely paid cash compared to those who were fully insured, i.e. $7,930.8 versus $1,958.1. Direct medical cost was highest among participants who were partially insured, amounting to $8,351.3, as they incurred expenses for both insurance premiums and hemodialysis sessions, which were not covered by their premiums. Participants who paid solely in cash had a direct medical cost of $5,851.3, followed by those who were partially exempted ($5,400.8). Participants who were fully insured had the lowest medical cost at $359.3, while participants who were fully exempted did not incur any direct medical cost.

**Table 4:** Cost of hemodialysis by payment modalities

By payment modalities	Mean (SD) (USD)	Median (IQR) (USD)
**Direct medical cost**		
Cash	5,851.3 (5,684.8)	4,494.4 (3,370.8–7,640.5)
Partially exempted	5,400.8 (4,796.9)	3,595.5 (2,022.4–8,089.8)
Fully insured	359.3 (237.3)	319.8 (194.5–648.2)
Partially insured	8,351.3 (2160.8)	9,312.9 (7065.6–9636.9)
**Direct non-medical cost**		
Cash	743.0 (1681.0)	224.7 (112.4–494.4)
Partially exempted	203.8 (273.1)	123.6 (78.7–191.0)
Fully exempted	786.5 (.)	786.5 (786.5–786.5)
Fully insured	682.7 (616.5)	449.4 (247.2–943.8)
Partially insured	449.4 (317.8)	337.1 (224.7–674.2)
**Indirect cost**		
Cash	1,336.4 (3965.3)	147.6 (147.6–888.9)
Partially exempted	490.2 (624.0)	147.5 (147.5–740.8)
Fully exempted	1,703.3 (.)	1,703.3 (1,703.3–1,703.3)
Fully insured	916.1 (2147.7)	147.6 (147.6–888.9)
Partially insured	1,185.0 (980.9)	1,258.8 (407.2–1,962.9)
**Average annual cost**		
Cash	7,930.8 (8041.3)	5,291.0 (4,010.9–9,300.9)
Partially exempted	6,094.9 (4805.3)	4,192.5 (2,449.4–8,730.3)
Fully exempted	2,489.8 (.)	2,489.8 (2,489.8–2,489.8)
Fully insured	1,958.1 (2244.6)	1,394.6 (920.3–2,265.1)
Partially insured	9,985.8 (1795.0)	10,281.8 (8,514.0–11,457.6)

Direct non-medical costs were more equally distributed among the payment modalities, except for the partially exempted and the partially insured. This is because all four partially insured participants reported that they did not have meals during treatment, while 30 (80%) out of 39 partially exempted participants used public transportation, which is cheaper, making the direct non-medical cost lower for these groups. Indirect cost was highest amongst participants who solely paid cash ($1,336.4), followed by those who were partially insured ($1,185.0). Those who were fully insured had an indirect cost of $916.1, while those who were partially exempted had the lowest indirect cost of $916.1.

### Cost of hemodialysis by the duration of treatment

Table 5 shows hemodialysis costs based on the duration of treatment. Participants who had been undergoing treatment for less than one year incurred slightly higher treatment costs, at $3,866.4, compared to those who had been in treatment for two to three years ($2,489.7) and those who had been in treatment for more than four years ($3,028.4).

The direct medical cost was highest among participants (*n* = 150) who had been in treatment for one year or less ($1,985.9), compared to those who had been in treatment for more than one year. This higher cost is attributed to a greater proportion of participants using cash payments in the first year (17%), compared to 10% for those in treatment for two to three years and 6% for those in treatment for four years or more. Additionally, in the first year, fewer participants reported having health insurance (73%) compared to 81.2% for those in treatment for two to three years and 79% for those in treatment for four years or more.

Participants who had been undergoing treatment for less than one year had an indirect cost of $1,204.6 compared to those who had been in treatment for two to three years ($644.3) and more than four years ($1,017.5). This is because 76 (51%) out of 150 participants who attended treatment for less than one year reported they could not work after starting treatment, while 102 (58%) out of 176 of those who had undergone treatment for two to three years reported they could not work after treatment and 75 (68%) out of 111 participants who had undergone treatment for four years and above reported that they could not work after starting treatment.

**Table 5:** Cost of hemodialysis in USD by duration of treatment

By Treatment Duration	Mean (SD)	Median (IQR)
**Less than 1 Year**		
Direct medical cost	1,985.9 (4,294.0)	432.1 (194.5–649.1)
Direct non-medical cost	675.9 (978.2)	376.4 (224.7– 898.9)
Indirect cost	1,204.6 (2549.2)	370.4 (147.6–1,444.6)
Average annual cost	3,866.4 (5294.2)	2,167.4 (1,059.4–4,010.9)
**2–3 Years**		
Direct medical cost	1,190.8 (2375.2)	432.2 (224.8–649.1)
Direct non-medical cost	654.6 (531.9)	449.4 (224.7–898.8)
Indirect cost	644.3 (1150.3)	147.6 (147.6–740.8)
Average annual cost	2,489.7 (2697.8)	1,482.3 (1,000.2–2,638.8)
**4 Years and Above**		
Direct medical cost	1,422.3 (2,907.7)	432.1 (216.1–649.1)
Direct non-medical cost	588.6 (905.6)	359.5 (179.8–719.1)
Indirect cost	1,017.5 (3257.8)	147.6 (147.6–814.3)
Average annual cost	3,028.4 (4,680.8)	1,366.2 (918.7–2,594.4)

### Cost of hemodialysis by the presence of comorbidities

Table 6 shows hemodialysis costs based on the presence of comorbidities. Participants with additional comorbidities such as cardiovascular diseases, hypertension, thyroid disease, cancer, and diabetes incurred a slightly higher average annual cost of $3,981.9 compared to those without comorbidities ($2,818.9). The direct medical, non-medical and indirect costs were also elevated among participants with comorbidities, amounting to $2,128.9, $784.3 and $1,068.7, respectively. In contrast, for participants without comorbidities, these costs were $1,373.9, $611.0 and $833.9, respectively.

**Table 6:** Cost of hemodialysis by the presence of comorbidities

By Presence of comorbidities	Mean (SD)	Median (IQR)
**Presence of Comorbidities**		
Direct medical cost	2,128.9 (4,899.2)	432.1 (155.5–656.8)
Direct non-medical cost	784.3 (1,343.4)	404.4 (157.3–898.9)
Loss in productivity	1,068.7 (2,256.2)	444.5 (147.6–1,258.8)
Average annual cost	3,981.9 (6,201.5)	2,275.5 (1,141.5–3,718.6)
**Absence of comorbidities**		
Direct medical cost	1,373.9 (2,745.3)	432.1 (216.2–649.1)
Direct non-medical cost	611.0 (603.1)	449.4 (224.7–898.9)
Loss in productivity	833.9 (2,369.8)	147.6 (147.6–740.2)
Average Annual cost	2,818.9 (3,641.9)	1,495.9 (930.6–3,001.5)

### Proportion of catastrophic health expenditure

91% of the patients incurred annual hemodialysis treatment costs that exceeded 10% of their income i.e., catastrophic health spending. This proportion decreased to77% when the 25% threshold of their household’s annual income was used. The proportion of CHE was higher among patients without health insurance compared to those who were fully covered i.e., 92% versus 73% at 25% threshold.

### Factors associated with the patients’ cost of hemodialysis

Table 7 presents the factors associated with the cost of hemodialysis. Income, payment modalities, duration of hemodialysis, and the number of hemodialysis sessions were significantly associated with the cost of hemodialysis. Similarly, there was no statistical differences in total average cost between private and governmental-run facilities.

**Table 7:** Factors associated with patients’ cost for hemodialysis

Average annual Cost	Coefficient	std. err.	*P* > z	[95% conf. interval]
**Income**				
$0	-0.01	0.115	0.941	-0.23–0.21
$43.6 - $86.4	0.05	0.203	0.812	-0.35 –0.44
$86.9– $129.6	0.13	0.191	0.484	− 0.24 – 0.51
$130.0 – $172.8	-0.03	0.181	0.879	− 0.38 – 0.33
$173.3 – $216.0	0.30	0.186	0.105	− 0.063 – 0.67
≥$216.0	0.77**	0.207	0.000	0.37–1.18
**Education**				
Higher Education	-0.12	0.215	0.569	− 0.54 – 0.29
Secondary Education	-0.21	0.241	0.388	− 0.68 – 0.26
Primary Education	-0.31	0.219	0.158	− 0.74 – 0.12
**Patient Sex**				
Female	-0.04	0.047	0.352	− 0.136 – 0.049
**Facility ownership**				
Government	-0.11	0.109	0.297	− 0.33 – 0.10
**Duration of hemodialysis**				
2–3 years	-0.23**	0.047	0.000	− 0.32 – − 0.14
4 years and above	-0.13	0.111	0.216	− 0.36 – 0.08
**Hemodialysis sessions**				
Two sessions	1.15**	0.218	0.000	0.72–1.58
Three sessions	1.61**	0.244	0.000	1.13–2.09
**Age**				
21–40 years	-0.20	0.238	0.400	− 0.67 – 0.27
41–60 years	-0.04	0.254	0.863	− 0.54 – 0.46
above 60 years	-0.35	0.217	0.103	− 0.78 – 0.07
**Treatment Payment**				
Cash	1.76**	0.106	0.000	1.56–1.97
Partial exempted	1.77**	0.123	0.000	1.53–2.01
Fully exempted	0.82**	0.172	0.000	0.48–1.15
Partial insured	1.25**	0.318	0.000	0.63–1.88
**Presence of comorbidities**				
No	0.05	0.079	0.561	− 0.11 – 0.20
_cons	6.23	0.489	0.000	5.27–7.19
/logs	-0.51	0.026		− 0.57 – − 0.46

**Note: Statistically significant at a p-value of less than 0.05

Regarding income, participants with a monthly income of more than or equal to $216.0 had hemodialysis costs reduced by a factor of 0.77 compared to those with a monthly income between $1 and $43. This is the only income level with significantly different hemodialysis costs relative to the monthly income level between $1 and $43.

When patients undergo hemodialysis for two to three years, the cost decreases by a factor of 0.23 compared to those who have undergone treatment for one year or less. The cost of hemodialysis increases by a factor of 1.14 for participants who attended two hemodialysis sessions compared to those who attended one session. For those attending three sessions per week, the cost increases by a factor of 1.61 compared to those attending one session.

Participants who paid fully in cash experienced a cost increase by a factor of 1.76 compared to those who were fully insured. Those who were partially exempted had a cost increase by a factor of 1.77 compared to fully insured participants. Conversely, those who were fully exempted had a cost reduction by a factor of about 0.82 compared to fully insured participants. Participants with partial insurance experienced a cost increase of 1.26 times compared to those who were fully insured.

## Discussion

Our study identified several factors associated with the costs of hemodialysis for patients, including income above $216, treatment payment modalities (such as cash, full exemption, partial exemption, and partial insurance), as well as the duration and number of hemodialysis sessions. Similarly, studies conducted in Burkina Faso and Malaysia found that income was the primary factor influencing treatment costs, with additional factors including age and the type of health facility [8, 14].

This study reported an average annual hemodialysis cost of $3099, which imposes a significant economic burden for patients in Tanzania, where more than a quarter (26.4%) of its population is living below the basic needs poverty line [29]. Almost 50% of the hemodialysis costs were direct medical costs averaging $1522.5 annually, and nearly a quarter were direct non-medical costs averaging $645.1 annually. Direct medical costs were incurred on consultation, laboratory tests, hemodialysis sessions and medicines, while direct non-medical costs were for transportation and meals. Tanzania is not the only country that faces high costs related to dialysis, as high patient costs of hemodialysis are reported globally. For instance, it is reported that, the average annual direct cost was $28,280 in the Democratic Republic of Congo [10], $1613 ($134.41 monthly) in Burkina Faso [8], and $3859 in Sudan [9].

The variation in reported costs across different countries can be attributed to several factors. Firstly, the components included in the cost calculations and the prices of consumables and services, such as medicines and fuel, differ. For example, the study in Burkina Faso included consultation, para-clinical examinations, hospitalization, medicines, and consumables as direct medical costs, and transportation, food, fuel, communication, and miscellaneous charges as direct non-medical costs [8]. Similarly, the study in Sudan included medication, investigations, doctor’s visits, and vascular access as direct medical costs, and transport, meals, and home changes as direct non-medical costs [9]. In contrast, this study included consultation, investigation fees, hospitalization costs, medicine costs, and insurance premiums as direct medical costs, and transportation and food costs as direct non-medical costs.

Secondly, the costing methods may differ between studies. Our study followed the WHO patient perspective costing framework, but the reviewed studies did not indicate whether they used the same framework or not. Also, some studies may mix patient costs with the providers’ costs, while our study only considered the out-of-pocket costs incurred by patients. For example, studies conducted in the Democratic Republic of Congo and Brazil considered direct medical costs from a hospital perspective [10, 30].

Thirdly, differences in the organization of healthcare systems and the complexity of facilities where these studies were conducted may also contribute to the variation in costs. The healthcare systems and facilities in these countries are different from those in Tanzania and the facility where this study was conducted.

This study investigates the impact of various treatment payment modalities on patients’ economic burden. The findings indicate that approximately 78% of participants were fully insured, incurring an average annual cost of $1,958, which is four times lower than the cost for patients who paid entirely out-of-pocket ($7,930.8). Similarly, a study conducted in Sudan reported higher out-of-pocket medical expenditure for patients without health insurance ($4,272.4) compared to insured patients ($1,634.7) [9]. Furthermore, our study found a substantial proportion (91%) of patients suffered catastrophic health expenditure. These results are similar to the ones reported in India, where between 95% and 96% of CKD patients on hemodialysis faced CHE [12, 13]. Our findings also show that patients who paid out of pocket were more prone to CHE compared to those with health insurance, i.e., 92% versus 73% at a 25% threshold. These results are in line with a study conducted in India, which reported that among 96% of CKD patients who faced CHE, 86% paid through out of pocket [13]. These findings demonstrate that patients paying out-of-pocket face a higher economic burden and underscore the importance of healthcare insurance in protecting patients, particularly those with chronic non-communicable diseases, from catastrophic health expenditure.

Supporting our indirect costs findings of $931.4 (30% of the average annual cost) are other studies that have also reported substantial productivity losses associated with hemodialysis. In India, the six-month cost due to wage loss during hemodialysis was estimated at R.77,726.7 ($1,893 per year) [31]. In Greece, productivity loss was estimated at €9.9 million ($15.5 million) [32], while it was reported at $1,025 and €1,300 annually in Ethiopia [33] and in the Netherlands [34] respectively. Additionally, a systematic review of employment among dialysis and kidney transplant patients reported a 16.4% decrease in employment rates for dialysis patients [35], while a European survey further revealed that 54.4% of dialysis patients had impaired physical ability to work, compared to 33.3% of kidney transplant patients [36]. The loss of working ability caused by hemodialysis can further lead to financial distress, which may result in underutilization of healthcare services and loss of follow-up during treatment [14, 37].

### Strengths and limitations

First, our research was conducted in the Dar es Salaam region, which has over 66% of the country’s CKD patients [18]. Secondly, we included both public (including the national hospital with a diverse patient population) and privately owned hemodialysis facilities, which may have provided a representative sample of CKD patients seeking hemodialysis therapy in the country. Thirdly, to compare their economic burden, we included all patients seeking hemodialysis therapy at the time of data collection, covering all payment modalities: cash, fully insured, partially insured, fully exempted and partially exempted. Fourth, to the best of our knowledge, no research has been undertaken to assess the economic burden of hemodialysis from the patient’s perspective in Tanzania. These findings will be useful to policymakers in designing financing strategies to protect CDK patients from the impoverishing expenses.

However, our study had some limitations. We did not account for intangible expenses such as pain, suffering, and mental distress caused by the disease and its treatment. We also did not consider the loss in productivity for caretakers, which may impact the overall treatment cost. Additionally, although we incorporated variables for which reliable and complete data were available to evaluate determinants of hemodialysis cost, other important variables, such as distance to the facility and household economic shocks, have been shown in the literature to influence the cost of hemodialysis. The inclusion of these variables in future research would strengthen explanatory power and provide a more comprehensive understanding of the determinants of hemodialysis costs. Despite these limitations, the findings of the study are relevant for stakeholders, particularly policymakers, since it is the first to provide the economic burden of CKD from the patients’ perspective in Tanzania.

### Policy implications and recommendations

The findings of this study highlight the importance of costing and economic evaluation studies in informing policies and guiding advocacy efforts to improve access to hemodialysis and CKD care in general. This includes prioritizing the establishment of a functioning and affordable health insurance scheme to reduce financial distress for hemodialysis patients in the country. Additionally, economic evaluation studies are beneficial in determining the best dialysis options given the available resources. Furthermore, establishing income protection insurance or aid for patients on hemodialysis who cannot work due to ill health can reduce financial stress for patients and their families and improve access and health outcomes.

## Conclusion

The annual cost of hemodialysis for CKD patients significantly exceeds the national minimum monthly wage for formal employees, placing a substantial strain on households, the health system and the country as a whole. Approximately 91% and 77% of patients incurred annual hemodialysis treatment costs that exceeded 10% and 25% of their household’s annual income, respectively. These catastrophic expenditures may drive patients and their families deeper into impoverishment and poverty. Additionally, hemodialysis treatment adversely affects labour market performance by causing significant productivity losses due to absenteeism and the inability to work caused by ill health.

## Supplementary Information

Below is the link to the electronic supplementary material.

Supplementary Material 1

## Data Availability

The datasets and materials used and/or analyzed in our study are available from the corresponding author upon request.

## Abbreviations

CHE: Catastrophic Health Expenditure
CKD: Chronic Kidney Disease
DALYs: Disability-Adjusted Life Years
GLM: Generalized Linear Model
IHME: Institute for Health Metrics and Evaluation
NHIF: National Health Insurance Fund
ODK: Open Data Kit
TZS: Tanzania Shillings
