# Supplementary material for: Economic burden of hemodialysis among patients with chronic kidney disease in Dar es-Salaam region in Tanzania - a cross-sectional study
Source: Cost Eff Resour Alloc. 2026 Apr 30;24:76. doi: 10.1186/s12962-026-00749-6 (PMC13289129; doi:10.1186/s12962-026-00749-6)
Supplement: Supplementary file 1 — Supplementary Material 1 [file 12962_2026_749_MOESM1_ESM.docx]

**Appendices:**

**Supplementary File 1**

**Table A1: Selected Hemodialysis Centres in Dar es Salaam:**

| **No.** | **Name of Hemodialysis Center** | **Ownership** | **Number of registered patients** | **Number of patients interviewed** |
| --- | --- | --- | --- | --- |
| 1. | Muhimbili National Hospital (Upanga) | Public | 200 | 167 (38.3%) |
| 2. | Muhimbili National Hospital (Mloganzila) | Public | 90 | 62 (14.2%) |
| 3. | Amana Reginal Referral Hospital | Public | 20 | 25 (5.7%) |
| 4. | CCBRT Hospital | Public | 35 | 22 (5.1%) |
| 5. | Shree Hindu Mandal Hospital | Private | 90 | 63 (14.5%) |
| 6. | Shree Hindu Mandal Super Specialized Polyclinic - Kunduchi | Private | 31 | 28 (6.4%) |
| 7. | Cardinal Rugambwa Hospital | Private | 47 | 35 (8%) |
| 8. | Baraka Dialysis Center | Private | 40 | 34 (7.8%) |

**Supplementary File 2**

**Table A2: Cost Category and Components**

| **Cost Category** | **Cost Component** |
| --- | --- |
| Direct Medical Cost | Consultation |
|  | Investigation (laboratory tests) such as hemoglobin test, Urea reduction ratio (URR) test, dialysis adequacy *(Kt/V) test.* etc |
|  | Medicines and medical supplies, such as erythropoiesis-stimulating agents (ESAs), supplements, and medicines bought for other existing comorbidities, such as hypertension, diabetes, etc. |
|  | Hemodialysis session – cost per hemodialysis session |
|  | Insurance premium |
| Direct non-medical cost | Transportation fee/cost - for patients and caretakers to and from the hospital |
|  | Meals when seeking treatment |
| Indirect Cost | Patient’s productivity loss |
